# Pathogenic somatic alterations of DDR genes in lung cancer are significantly different from germline mutations and are associated with more unstable genomes

**DOI:** 10.1186/s12967-022-03577-3

**Published:** 2022-09-06

**Authors:** Huaqing Wang, Yue Zhao, Fei Wang, Xiaofeng Zhu, Ningning Luo, Tingting Sun, Chuang Qi, Xin Li

**Affiliations:** 1 Department of Oncology, Tianjin Union Medical Center of Nankai University, Tianjin, China; 2 Department of Oncology, Qinhuangdao Fourth Hospital, Qinhuangdao, China; 3 The Medical Department, Jiangsu Simcere Diagnostics Co., Ltd, Nanjing Simcere Medical Laboratory Science Co., Ltd, The State Key Lab of Translational Medicine and Innovative Drug Development, Jiangsu Simcere Diagnostics Co., Ltd, Nanjing, China; 4 Cardiothoracic Surgery Department, Tianjin Medical University General Hospital, Tianjin, China

To the Editor

The defects DNA-damage repair (DDR) genes would drive tumor formation and are associated with increased genomic instability and tumor mutational burden (TMB) in cancer [1]. Although, alterations of DDR genes are common in NSCLC, the differences between the germline and somatic alterations are poorly characterized.

The DNA sequencing data of 540 genes from 5235 lung cancer patients were retrospectively collected and 276 DDR genes were analyzed [2]. The variations were annotated as pathogenic (P), likely pathogenic (LP), and non-pathogenic (NP) according to ACMG (American College of Medical Genetics) guideline. The patients were divided into 3 groups (Table 1): DDR-germline (P&LP germline variants, N = 650), DDR-somatic group (only P&LP somatic variants, N = 1489) and the non-DDR group (NP variants, N = 3096). The DDR-somatic group had a higher median age and the highest proportion of males, stage IV and LUSC patients.

**Table 1:** Comparison of clinical data for NSCLC patients in non-DDR, DDR-somatic, and DDR-germline groups

Characteristics	N	Level	non_DDR N (%)	DDR_somatic N (%)	DDR_germline N (%)	P value
Total	5235		3096	1489	650	
Age	5232		62 (16–94)	64 (23–107)	62 (27–92)	1.43E−07
Gender	5235	Female	1590 (51.36%)	554 (37.21%)	293 (45.08%)	1.9443E−18
		Male	1506 (48.64%)	935 (62.79%)	357 (54.92%)	
Stage	2380	I	255 (19.26%)	48 (6.43%)	48 (15.48%)	5.2446E−13
		II	129 (9.74%)	59 (7.91%)	29 (9.35%)	
		III	242 (18.28%)	157 (21.05%)	64 (20.65%)	
		IV	698 (52.72%)	482 (64.61%)	169 (54.52%)	
Dignosis	3888	NSCLC	2266 (98.65%)	1075 (96.85%)	475 (98.75%)	0.00071134
		SCLC	31 (1.35%)	35 (3.15%)	6 (1.25%)	
Subtype	3544	LUAD	1923 (91.22%)	827 (82.95%)	382 (87.02%)	1.0291E−09
		LUSC	148 (7.02%)	140 (14.04%)	50 (11.39%)	
		Others	37 (1.76%)	30 (3.01%)	7 (1.59%)	

The most commonly germline alterations were found in BRCA2 (8.46%), ERCC2 (8.15%) and IDH1 (8%), while in the somatic mutations, TP53 (89.05%) showed the highest frequency (Fig. 1A, B). Among the ten functional categories of DDR genes, the mutations of Fanconi anemia (FA) (234, 36.00%) and homology-dependent recombination (HR) (249, 38.31%) signaling were enriched in germline, while other categories (1417, 95.10%) were more common in somatic alterations.

**Fig. 1:**
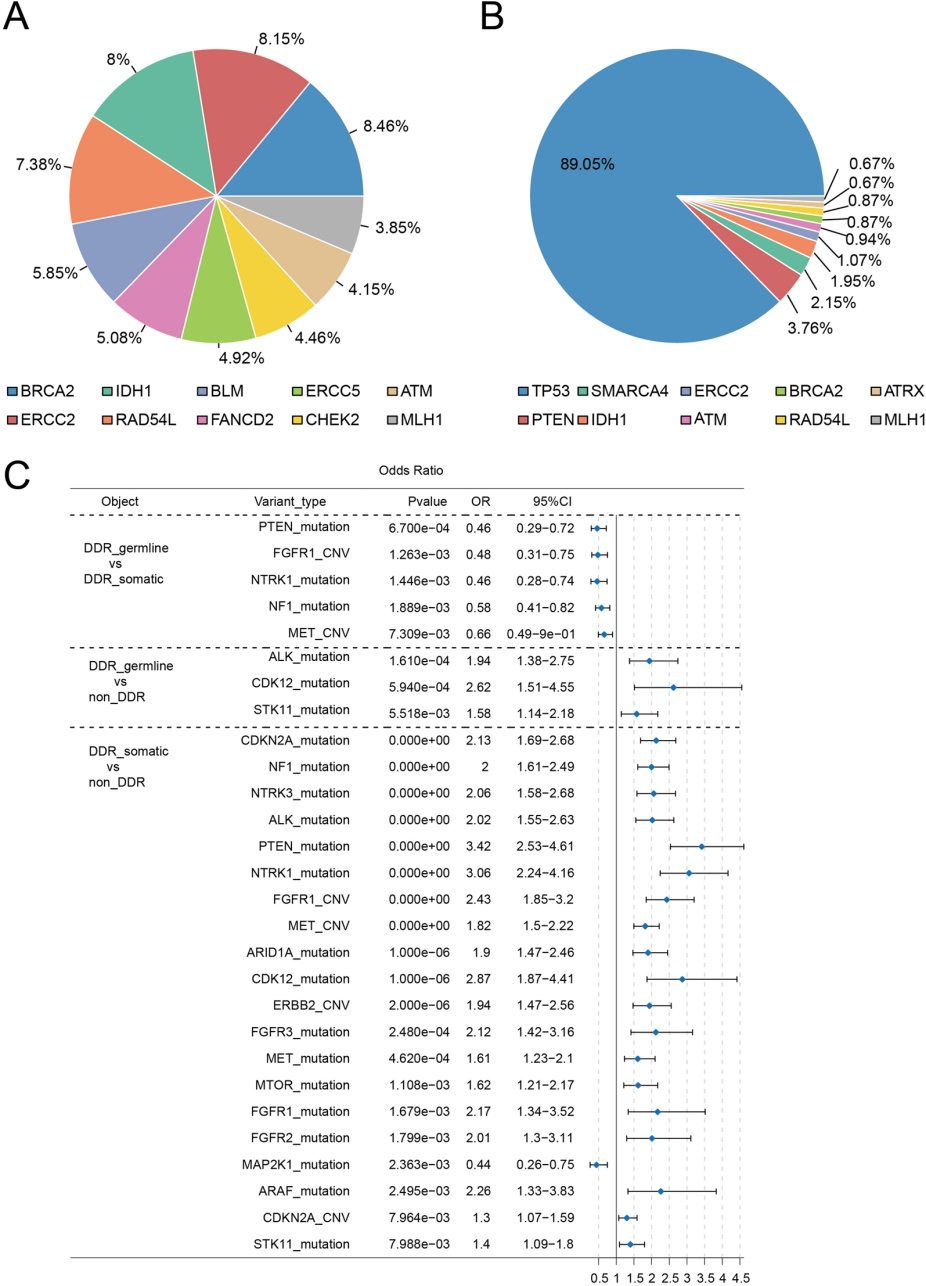
DDR gene mutation landscapes of the DDR-germline (**A**) and DDR-somatic (**B**) groups and the comparation of the alterations in actionable genes among the three groups (**C**)

In pairwise comparisons of the three groups, 28 actionable mutations were assessed based on OncoKB [3]. DDR-somatic group was more likely to have alterations in *PTEN* (OR = 0.46), *FGFR1* (OR = 0.48), *NTRK1* (OR = 0.46) compared with DDR-germline group. Taken non-DDR group as reference, mutations in *ALK* (OR = 1.94), *CDK12* (OR = 2.62), *STK11* (OR = 1.58) were more common in DDR-germline group, but *CDKN2A* (OR = 2.13), *NF1* (OR = 2), *NTRK3* (OR = 2.06) in DDR-somatic group (Fig. 1C).

The tumor mutational burden (TMB) was statistically different among the three groups (P < 2.22e−16). The DDR-somatic group exhibits the highest median TMB (12.39) compared with 7.44 in DDR-germline and 5.24 in non-DDR group. In addition, the proportion of MSI-H patients in DDR-somatic group is the highest (0.94%) compared with 0.62% in DDR-germline group and 0.23% in non-DDR group (P = 0.0040) (Fig. 2A).

**Fig. 2:**
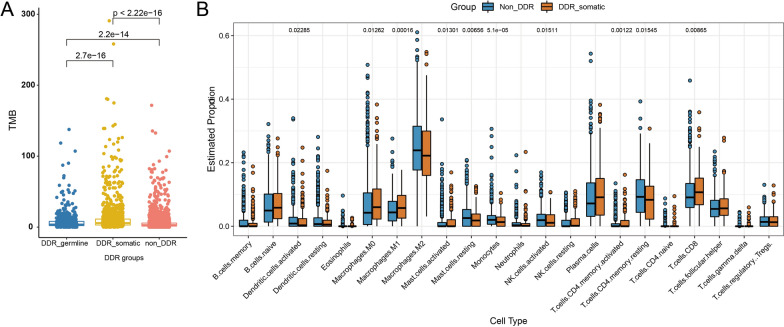
The differences of TMB in the DDR-germline, DDR-somatic and non-DDR groups (**A**) and the immune microenvironment assays in the DDR mutation and wild type groups (**B**)

The genetic data of 1053 lung cancer patients were downloaded from TCGA. The TCGA cohort was stratified into DDR-somatic and non-DDR groups according to the pathogenic annotation by Clinvar. Ten types of immune cells were found significantly associated with DDR status (Fig. 2B), Macrophages M0 (P = 0.0126), Macrophages M1 (P = 0.0002) and CD8 T cells (P = 0.0087) showed higher proportions in DDR-somatic group.

The differences in the mutation profile between the DDR-germline and DDR-somatic groups and the distinct actionable genes may indicate the different target-therapy choices for NSCLC patients. Besides, patients with somatic pathologic mutations exhibit the highest genome instability, including the highest TMB and the most MSI-H, and superior immune microenvironment consist of higher proportions of macrophages and CD8 cells infiltration. According to a previous report, patients with pathologic DDR mutations had higher objective response rate, longer median progression-free survival and overall survival with PD-L1 therapy [1]. This may indicate patients with somatic DDR alterations may better benefit from the immune checkpoint inhibition in NSCLC.

## Data Availability

The datasets used and/or analysed during the current study are available from the corresponding author on reasonable request.
